# In-Line Phase-Contrast X-ray Imaging and Tomography for Materials Science

**DOI:** 10.3390/ma5050937

**Published:** 2012-05-24

**Authors:** Sheridan C. Mayo, Andrew W. Stevenson, Stephen W. Wilkins

**Affiliations:** CSIRO Materials Science and Engineering, Private Bag 33, Clayton, VIC 3169, Australia; E-Mails: andrew.stevenson@csiro.au (A.W.S.); steve.wilkins@csiro.au (S.W.W.)

**Keywords:** phase-contrast, micro-tomography, X-ray imaging, radiography, X-ray microscopy

## Abstract

X-ray phase-contrast imaging and tomography make use of the refraction of X-rays by the sample in image formation. This provides considerable additional information in the image compared to conventional X-ray imaging methods, which rely solely on X-ray absorption by the sample. Phase-contrast imaging highlights edges and internal boundaries of a sample and is thus complementary to absorption contrast, which is more sensitive to the bulk of the sample. Phase-contrast can also be used to image low-density materials, which do not absorb X-rays sufficiently to form a conventional X-ray image. In the context of materials science, X-ray phase-contrast imaging and tomography have particular value in the 2D and 3D characterization of low-density materials, the detection of cracks and voids and the analysis of composites and multiphase materials where the different components have similar X-ray attenuation coefficients. Here we review the use of phase-contrast imaging and tomography for a wide variety of materials science characterization problems using both synchrotron and laboratory sources and further demonstrate the particular benefits of phase contrast in the laboratory setting with a series of case studies.

## 1. Introduction

The origins of X-ray tomographic methods sit firmly in the medical sphere with the development of computerized tomography (CT) by Hounsfield and Ambrose in the early 70s [1,2]. Only a few years later the first forays into micro-CT were made, primarily for biomedical applications [3,4,5,6], including at synchrotron sources [7]. Since these early times micro-CT has increasingly been incorporated into the microscopist’s toolkit for 3D characterization of materials [8,9,10,11,12], a development which has been greatly assisted by the increasing availability of turnkey laboratory X-ray micro-CT systems [13,14].

Conventional tomographic methods all have the common feature that they rely solely on the absorption of X-rays as a mechanism of contrast formation. However, around the time of the development of lab-based micro-CT systems in the mid 60s, the first steps were being taken to create X-ray images using the refraction of X-rays by matter, in addition to absorption. These types of refractive-, or phase-contrast-, imaging methods enable the visualization of features in weakly absorbing objects such as polymers, or indeed objects that barely absorb X-rays at all. They also greatly enhance the visibility of weakly absorbing features in the presence of more strongly absorbing sample components, for example enabling thin filamentary components such as carbon fibers to be clearly imaged in a sample that also contains metals.

X-ray phase-contrast imaging was first deliberately implemented with interferometric methods in which a reference beam is allowed to interfere with a beam, which has passed through the sample [15,16,17]. A second class of phase-contrast imaging approaches are analyzer-based imaging methods involving reflection of the transmitted beam from a Bragg crystal which acts as an angular filter such that refractive effects caused by the object are converted into intensity effects in the detector plane. These would appear to have first been developed by Goetz and co-workers [18,19] in the context of imaging the internal structure of pellets for use in thermonuclear fusion experiments. In the case of condenser-objective type X-ray microscopes, Zernike phase-contrast methods have also been implemented in a similar manner to that for their visible light counterpart for both diffractive and refractive optics [20,21,22,23]. For scanning transmission X-ray microscopes a segmented detector can be used to detect the refraction (deflection) of the probe beam by the sample, a method, which has been implemented both at synchrotrons and in laboratory systems [24,25,26]. A more recent class of approaches to phase-contrast imaging is based on the use of one or more gratings to act as wave-front modulators and/or analyzers [27,28,29]. A further class of methods includes those termed Coherent Diffractive Imaging, which seek to exploit highly coherent beams to obtain coherent diffraction patterns from small samples or small regions of a sample, enabling very high-spatial resolution information to be extracted [30,31,32]. A number of these phase-contrast methods have been applied in materials micro-tomography [33,34,35,36,37,38,39].

The simplest X-ray phase-contrast method, and the one which is the main focus of this paper, follows in the footsteps of Gabor’s development of in-line holography for improving the resolution of electron microscopy [40]. This technique, better known as in-line phase-contrast, makes use of the Fresnel diffraction of X-rays to enhance the visibility of edges and boundaries within an object and was first observed in the holographic imaging regime using soft X-rays from a synchrotron source [41]. In-line phase-contrast was first demonstrated with monochromatic hard X-rays at synchrotron sources by Snigirev *et al*. [42] and independently using polychromatic laboratory sources by Wilkins *et al*. [43,44]. The work and research that followed clearly demonstrated the potential of the in-line method for imaging weakly absorbing features in low-density objects [45,46,47]. The technique has been widely applied in synchrotron tomography [48,49] and lab based imaging and tomography with microfocus sources [50,51] and specially adapted scanning electron microscopes (SEMs) [52,53]. 

The key enabling property for high quality in-line X-ray phase-contrast imaging is high lateral spatial coherence of the source as viewed from the sample, where lateral spatial coherence is defined by Equation 1.

(1) $$l_{c}=\lambda \;\frac{R_{1}}{2\pi \sigma }$$

where *σ* denotes the rms X-ray source size and *λ* the X-ray wavelength [54].

For conventional laboratory sources this is commonly (but not always) achieved by the use of a microfocus source of, say, 25 μm or less (FWHM). For synchrotron sources, high lateral spatial coherence is commonly achieved either by having a long path length between the primary X-ray source (inside the storage ring) and the sample, or by the use of some form of focusing optics to produce a small secondary effective source. For imaging under near-field conditions (first Fresnel zone), chromatic/spectral coherence is not important and a wide band-pass of an order of 30% is typically acceptable [44,55]. The advantages of laboratory sources for in-line phase-contrast imaging are; Stability, possibility of large magnification and hence the ability to use comparatively low spatial resolution detectors (such as Imaging Plates or flat panel detectors) and the possibility to use energy-resolving detectors to do multi-spectral imaging. The latter are not so readily applied at synchrotron sources due to the much high intensities involved. On the other hand, the principal advantage of synchrotron sources for X-ray in-line phase-contrast imaging is a very high intensity, which makes dynamic studies possible. 

More quantitative approaches have incorporated phase-retrieval methods, which enable the extraction of the phase-shift imposed on the X-ray wave-front by the object [56,57]. These methods were soon extended to tomography [58], and also to more absorbing samples [59,60,61]. Phase-retrieval was also extended to multi-distance and multi-energy data sets with benefits for the analysis samples composed of multiple materials [62,63].

With the increased availability of micro-focus X-ray sources in-line phase-contrast imaging is now much more accessible to researchers, indeed with very small micro-focus sources (say 5 μm FWHM or even less) operating at high magnifications it is almost unavoidable. A good understanding of the optimal application of these methods for tackling problems in materials characterization is therefore highly desirable. This paper aims to provide an overview of the use of phase-contrast imaging and tomography with a review of the state-of-the-art of in-line phase-contrast imaging and tomography for materials characterization both at synchrotrons and in the laboratory. It will also illustrate the capability of the technique with a series of case studies demonstrating the application of lab-based in-line phase-contrast methods to a variety of materials applications.

## 2. Review

### 2.1. Physical Principle of In-Line Phase-Contrast Imaging

**Figure 1:**
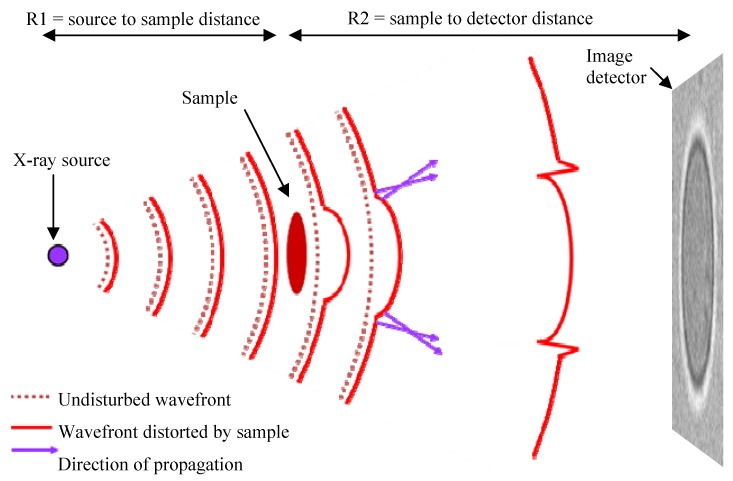
Mechanism of in-line phase-contrast: A sample imposes a phase-shift on the X-ray beam, which distorts the X-ray wavefront.

In-line phase-contrast imaging, like other phase-contrast methods, is made possible by the refraction of X-rays by a sample. The X-ray refractive index is very close to 1, making these refractive effects weak compared to the refraction of light, therefore specialized imaging conditions are required to make use of X-ray refraction in imaging. The primary requirement is that the X-ray beam illuminating the sample has high spatial coherence; a condition that is met either by having the X-ray source some distance away, as is typically the case at a synchrotron, or by using a small source size (of a few tens of microns or less). The second significant requirement is that there is a significant distance between the sample and the detector. After passing through the sample the X-ray wavefront is distorted in proportion to the phase-shift imposed by the sample, and it is the propagation of the distorted X-ray wavefront between the sample and detector which gives rise to Fresnel diffraction fringes in the image. An important feature for practical applications is that there is no strong requirement for chromatic coherence since in the near-field case the Fresnel fringes are approximately coincident for different energies, enabling polychromatic radiation such as that from laboratory sources to be used [44]. Figure 1 shows the in-line phase-contrast mechanism for the cone-beam imaging geometry typical of X-ray micro-CT experiments.

This gives rise to small changes in propagation direction of adjacent parts of the wavefront leading to interference fringes at object boundaries in the image. This illustration is for the cone-beam geometry typical for lab-based imaging. At a synchrotron the wavefront is approximately planar but the image forming mechanism is essentially the same.

In the parallel beam case the intensity of the phase-contrast fringes increases with the propagation distance from sample to detector *R*_2_, although as this distance increases further the fringes broaden and the imaging regime changes from the near-field, edge-enhanced mode used in most phase-contrast imaging to the intermediate regime where more diffraction fringes are visible, e.g., as used in holotomography [62]. 

In the cone-beam case the image of the sample is magnified on the detector according to the geometry such that magnification *M* = (*R*_1_ + *R*_2_)/*R*_1_, where *R*_1_ and *R*_2_ are the source-to-sample and sample-to-detector distances, respectively. Assuming that we have a point source and perfect detector, it can be shown that the phase-contrast fringes will be strongest where *R*_1_ = *R*_2_ (*i*.*e*., a magnification of 2), however, in practice detector resolution restricts the imaging geometries in which fringes can be observed to higher magnifications where *R*_1_ < *R*_2_ which also corresponds to the typical set-up for micro-CT of small samples.

If we ignore the effect of source size and finite detector resolution, for X-rays of wavelength *λ* the spacing of the phase-contrast fringes relative to the sample will be *S_F_ = √(R’λ*) where *R’* = *R*_1_*R*_2_/(*R*_1_ + *R*_2_). They will be visible (e.g., phase-contrast imaging will be possible) where they can be resolved by the imaging system, which will depend on the magnification, source size and detector resolution. This is reasonably easy to achieve with modern microfocus X-ray sources of <10 μm in source-size and CCDs of pixel size 20 μm or less. A detailed discussion of contrast, resolution and optimization of imaging conditions for in-line phase-contrast imaging can be found in several papers [64,65,66,67].

### 2.2. Phase-Retrieval for Quantitative Phase-Contrast Imaging

Phase-contrast images, as noted above, are edge enhanced so that the image intensity is no longer linked in a straightforward way to the thickness of the sample. In a phase-contrast image both the brightest and lowest intensities are likely to be found in the fringes delineating the edges and any internal boundaries of the sample. In order to extract quantitative information from an image a phase-contrast image can be transformed by an operation called phase-retrieval to recover the original phase-shift imposed on the X-ray wavefront by the sample (e.g., immediately after the sample). A phase-retrieved image is not only a more quantitative representation of the sample, it is better suited as input to a tomographic reconstruction algorithm.

A significant proportion of phase-retrieval algorithms used in the context of in-line phase-contrast imaging are based on the transport of intensity equation which describes how the intensity distribution of a wave evolves as it propagates [68,69]. This approach was first used in the context of hard X-ray imaging by Nugent *et al*. [57] who applied it to synchrotron-based imaging of pure phase objects. Application of this type of technique to polychromatic laboratory data was demonstrated by Gureyev and co-workers [70]. Bronnikov showed that for tomography of pure phase objects this type of phase-retrieval method could be combined with the reconstruction step, thus speeding up computation [58]. 

The methods previously described are only strictly correct for a sample which does not absorb X-rays but only refracts them. Phase-contrast images of many samples however contain both phase and absorption contrast. For weakly absorbing samples, small modifications of the previous methods may suffice [60]. Algorithms for more strongly absorbing samples have also been developed and are widely used for phase retrieval from a single phase-contrast image [59,61,71,72,73,74]. In their practical implementation these latter algorithms are almost identical in that they only give strictly quantitative results for a monochromatic X-ray beam and a single material object (or more strictly speaking an object in which the ratio of the real and imaginary parts of the X-ray refractive index is constant), unless the X-ray energies are very high (>60 keV). Nevertheless they are widely and quite successfully used in practical imaging and tomography applications even outside their area of strict applicability, for instance for multi-material samples or polychromatic X-ray beams. A review of the use of many of these algorithms can be found in a recent paper by Burvall [75]. 

By incorporating prior knowledge about the sample it is possible to extend single-image phase retrieval to provide quantitative results for multi-material samples [76,77], or to speed up CT data collection by enabling fewer views to be used views [78]. In the case of polychromatic X-ray sources, such as those found in the lab, spectrally weighted averaging methods can be used for accurate phase-retrieval [79,80].

The previously described algorithms are restricted to the more typical “edge enhanced” form of in-line phase contrast, sometimes known as the “near-field” imaging regime which corresponds to short propagation distances between sample and detector. Phase-contrast imaging in the intermediate-field regime (*i*.*e*., with longer propagation distances) results in an image with more obvious diffraction characteristics due to the visibility of high-order Fresnel fringes. For images which do not depart too far from the near-field regime the Born and Rytov approximations can be used with TIE-based methods similar to those above [81]. For images with stronger diffraction features Cloetens’ holotomography method gives impressive results but requires multiple images at different propagation distances [62]. For weakly absorbing samples single image algorithms based on the extended contrast-transfer function are also effective [82,83].

### 2.3. Synchrotron In-Line Phase-Contrast for Materials Characterization

#### 2.3.1. Cracks/Defect Initiation in Various Materials

Phase-contrast imaging and tomography has made significant impact in the study of cracks and damage mechanisms in various materials making particular use of the high sensitivity to features such as voids, porosity, and even boundaries between quite similar materials [46]. This has been applied to the investigation of Al:SiC metal matrix composites (MMCs) by Buffiere and co-workers who were able to identify rupture of the SiC particles as the starting point of plastic deformation [84,85,86]. Growth defects in SiC such as micropipes have been examined using white-beam phase-contrast [87], sometimes paired with Bragg diffraction [88]. Phase-contrast micro-tomography has also been applied to the analysis of wetting characteristics of functionally graded Al/SiC MMCs [89]. These are produced by a casting process where imperfect wetting leads to the unintended incorporation of voids in the material.

Cracks in more conventional metals and alloys have been studied by Ignatiev and co-workers who used stereometry to map the 3D crack structure using pairs of 2D images [90]. Herbig analyzed 3D growth of fatigue cracks in the polycrystalline microstructure Ti 21 S by combining in-line phase-contrast imaging with diffraction contrast tomography to get both crack morphology and grain shape and orientation [91]. Dynamic studies of crack formation have also been reported. These include 3D crack tomography during *in situ* fatigue crack loading of Ti-6246 alloy [92] and 3D observation of damage evolution and calculation of local crack driving forces in aluminum alloys from *in-situ* phase-contrast micro-CT during load cycles [93,94,95].

There have been a number of studies of cracking in metallic foams. For instance, Koboyashi and Toda combined phase-contrast micro-CT with other techniques such as local micro-CT and *in-situ* methods to analyze cracks in foams of pure Al and Al alloys. They discovered that foam morphology and micro porosity were the main factors in ductile buckling (pure Al) and brittle fracture (alloy) of cell walls during compressive deformation [96,97,98]. Hu and colleagues [99] analyzed the nucleation of cracks in layered foams from the insulation on the space shuttle fuel tank. These experiments were performed with *in-situ* loading and showed that the interfaces between the layers and the interlayer bonding were the starting point for defects and cracks.

The analysis of crack formation and growth has also been extended to materials as diverse as bio compatible acrylic cements, where crack mechanisms have been linked to beads at surfaces of voids within materials [100] and mortars used in construction. A study of mortars affected by weathering via the alkali-silica reactions enabled observation of micro-cracking and gel deposition induced by an accelerated weathering process [101].

#### 2.3.2. Low Density Materials and Low-Contrast Boundaries

In addition to sensitivity to cracks and voids, phase-contrast imaging methods have also proven very valuable in a number of materials-science applications which benefit from its sensitivity to features in low-density materials, and to other low-contrast features such as boundaries between similar materials. Baruchel and colleagues demonstrated the ability to image low-contrast biofilms and thin hydroxyapatite layers using phase-contrast [102]. The technique has also been applied to the visualization of carbon fibers in carbon-carbon (C/C) composites, which requires sensitivity to a very small difference in X-ray refraction between the two materials [103,104]. Kobayashi and colleagues further extended this approach for a study of activated C/C composites used for electric-double-layer capacitor electrodes by incorporating a Bragg magnifier to increase resolution and enable real-time imaging of the behavior of the composite within an overcharging capacitor [105]. 

Other composite also provide challenges to absorption-based X-ray imaging and tomography. Phase-contrast has been utilized for analysis of fiber orientations of carbon fibers in polymer matrices [106] and for characterization of composites with CaCO_3_ fillers in the matrix reinforced with glass fibers [107]. Young and co-workers combine phase-contrast micro-CT analysis of structural deformation with diffraction analysis of lattice strains to explore the mechanical behavior of interpenetrating Al_2_O_3_/Al composites which also have low-contrast boundaries [108].

Low-density materials have found potential applications in nuclear technology including the pyrocarbon and silicon carbide coatings for use in fuel pellets for fusion reactors. Kashyap and co-workers have analyzed the uniformity and quality of such coatings on test samples based on alumina and zirconia microspheres [109,110]. Even lower density materials are used in inertial confinement fusion capsules, which contain a solid deuterium-tritium layer in a copper doped beryllium shell and which have been usefully characterized using phase-contrast methods [111,112]. 

Phase-contrast micro-tomography of Al/Si alloys used in semi-solid forming has been demonstrated by Verrier and co-workers [113]. In this case the phase-contrast enables the different phases to be distinguished which would be impossible with absorption contrast alone as the attenuation coefficients for the different phases are very similar. This study enables the solid and liquid phases to be clearly distinguished and their connectivity determined.

#### 2.3.3. Porous Materials

The characterization of porous materials touches on a wide range of applications in materials science. Weiss *et al*. report on tomographic characterization of bone in-growth into porous bone repair materials [114]. Phase-contrast imaging has also made an impact here in the study of gas diffusion layers enabling the detailed characterization of morphology and the visualization of the water saturation as a function of pressure [115,116]. On a finer scale, submicron porosity in the form of tubules in tooth dentin has been imaged using specialized high sensitivity methods of in-line phase-contrast imaging [117]. There may be considerable future potential in using X-ray phase-contrast for the visualization of low-density fluids such as water in other types of porous materials including mortars and rocks.

#### 2.3.4. Natural Materials

Many natural materials such as wood and paper are composed of low Z elements which absorb X-rays only weakly and which benefit from the use of phase-contrast in imaging. Phase-contrast tomography was applied to paper samples relatively early in the development of in-line phase-contrast methods [118,119] and showed great promise for distinguishing low-density cellulose fibers. More recently it has been applied to analysis of morphological and transport properties in dry and soaked paper samples [120].

Wood, which has a similar composition to paper and also has an intricate hierarchical 3D structure, has been imaged using synchrotron phase-contrast by Groso and also Trtik and colleagues who were able to extract porosity and microstructural parameters such as cell-wall thickness with an imaging resolution down to 1.5 μm [60,121].

### 2.4. Laboratory-based In-Line Phase-Contrast for Materials Characterization

The development of lab-based in-line phase-contrast imaging has run in parallel to synchrotron methods due to the ease with which it can be applied using polychromatic lab sources. Unlike a synchrotron source where spatial coherence is usually provided by distance from the source to sample, lab-based phase-contrast typically makes use of micro-focus X-ray sources, which are available from a range of manufacturers. Lab sources, particularly micro-focus sources, have a much lower X-ray flux than synchrotron sources necessitating data collection times of hours rather than minutes. Nonetheless access to lab sources is much more readily available than synchrotron beam-time, and excellent phase-contrast results can be obtained. 

The earliest lab-based studies are reported by Wilkins and co-workers [44] who applied the technique to paper and wood [122,123]. Zoofan and colleagues give an overview of potential for non-destructive materials evaluation including its use in the imaging of corrosion pits and porosity in alloys [51,124]. Arhatari and co-workers develop a model for optimizing phase-contrast in a polychromatic system which they applied to imaging fine cracks in aluminum [125].

The application of phase-retrieval methods outside of the conditions in which they are formally valid has been to improve the quality of imaging for materials as is demonstrated for the case of Aerosil granule and a hydrated bentonite gel [125]. 

The benefit of phase-contrast in imaging low-density materials is demonstrated in a study of interfaces in graded aerogels with densities in the range of 20–200 mg/cm^3^ [126] and in imaging of deuterium-tritium fuel layer inside inertial confinement fusion fuel pellets [127].

Phase-contrast tomography has been applied successfully in a range of applications. These include the three dimensional characterization of woven composites by Djukic [50], analysis of filler dispersion in thermoplastic composites by Wu [128], and analysis of porosity in cold-sprayed titanium [129].

The following section highlights a number of case-studies from the authors’ work showing different aspects of the use of laboratory-based phase-contrast imaging applied to a wide range of materials characterization problems.

## 3. Applications of In-Line Phase-Contrast Imaging—A Selection of Materials Science Case Studies

### 3.1. In-Line Phase-Contrast Imaging with a Micro-Focus Source

In this section we will briefly demonstrate the utility of phase-contrast X-ray imaging with microfocus sources for certain materials-science applications. The X-ray source used is a Feinfocus FXE-225.20 tube with a cylindrical, reflection-geometry, W target and a 250 μm thick Be window. The minimum source size is approximately 4 μm; the maximum accelerating voltage 225 kVp; the maximum tube current 3 mA; and the maximum power 320 W. The source is mounted on a large optical table, disposed so that the optic axis is horizontal. The distance of closest approach to the X-ray spot is approximately 12 mm and the optical table provides for a maximum source-to-detector distance of around 2 m.

In the following sub-section we will provide examples of two-dimensional PCX imaging with image plates. Then in Subsection 3.1.2, we provide an example of PCX tomography with a CCD.

#### 3.1.2. PCX—Two-Dimensional Imaging

The Fuji image plates used in these studies are 20 cm × 25 cm in size and are scanned in a Fuji BAS-5000 scanner with a 25 μm pixel size selected. The plates used (FDL-URV) are specially formulated to achieve high spatial resolution (~100 μm), with some concomitant sacrifice of sensitivity (see also [63,64] for further details). The images presented in this sub-section are shown as negatives, *i*.*e*., dark regions signify higher X-ray exposure and light regions lower X-ray exposure.

**Figure 2:**
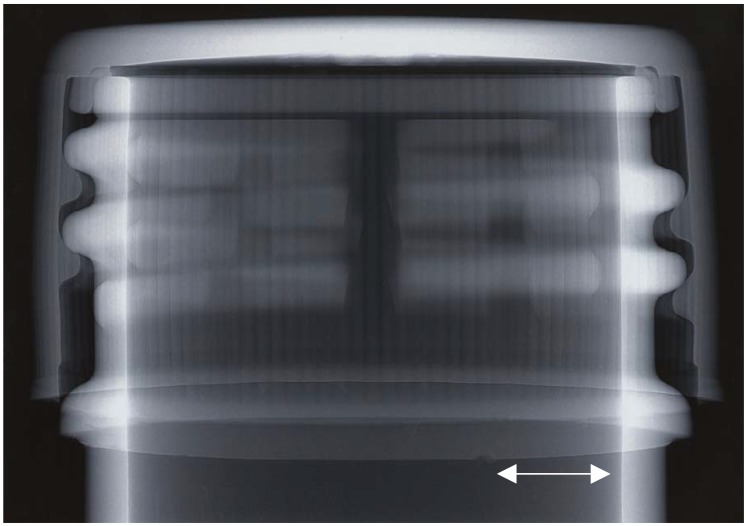
PCX image of the top of a plastic bottle, including cap: 30 kVp, 50 μA; source-to-sample distance = 20 cm; sample-to-detector distance = 80 cm; exposure time 45 s.

As a first, very simple demonstration of the use of a laboratory-based microfocus X-ray source to achieve edge enhancement via phase contrast, Figure 2 shows an image collected for the top of a plastic bottle (empty), with the screw cap in place. The X-ray source was operated at 30 kVp, 50 μA, with a source-to-sample distance of 20 cm and a sample-to-detector distance of 80 cm (experimental magnification of five). The exposure time was 45 s.

X-ray absorption is particularly obvious for the longer path-lengths through the plastic, such as at the top of the screw cap. Edge enhancement via phase contrast is also very clear, e.g., at the vertical striations on the screw cap. Phase contrast helps to delineate quite subtle features such as a small nick at the base of the screw cap (indicated by the vertical arrow).

**Figure 3:**
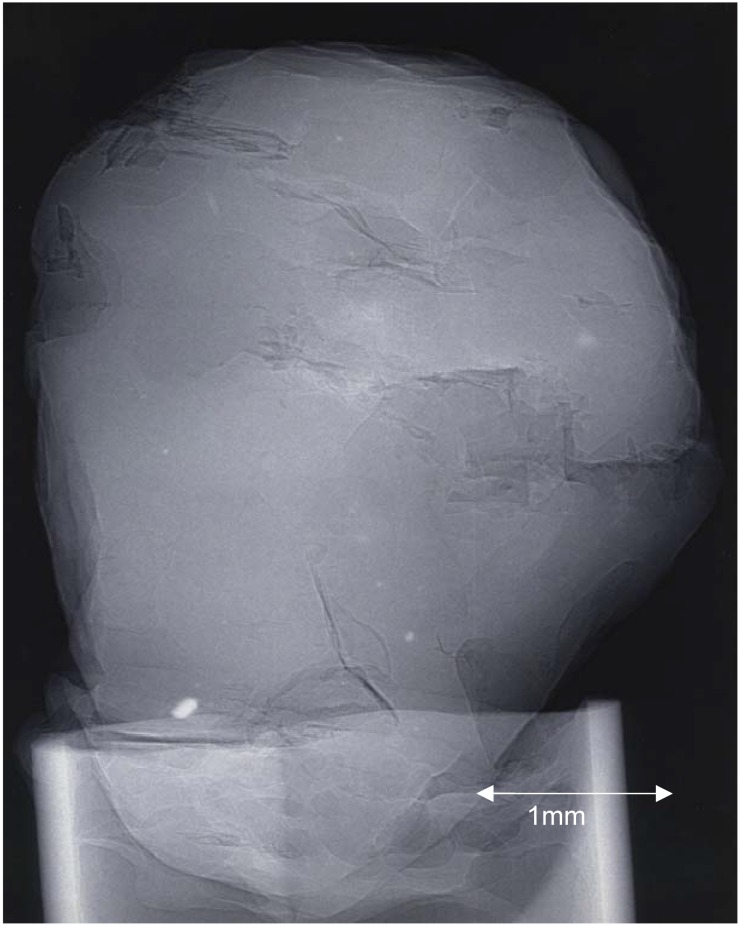
PCX image of a small industrial diamond: 30 kVp, 50 μA; source-to-sample distance = 10 cm; sample-to-detector distance = 190 cm; exposure time 120 s.

The second example (see Figure 3) is a small (~3 mm diameter) industrial diamond. The diamond has been mounted in a hollow glass tube. The X-ray source was operated at 30 kVp, 50 μA, with a source-to-sample distance of 10 cm and a sample-to-detector distance of 190 cm (experimental magnification of 20). The exposure time was 120 s. A number of features, defects and inclusions are clearly visible, some as a result of edge enhancement via phase contrast.

**Figure 4:**
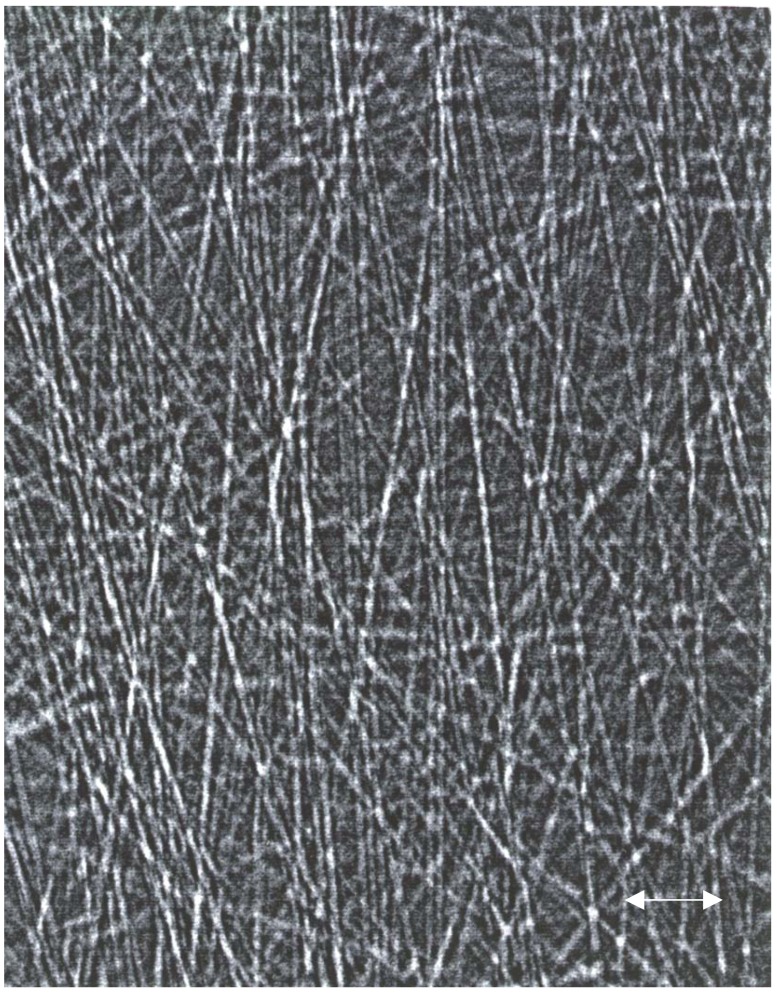
PCX image of a Toray carbon-paper sample (approximately 100 μm thick): 30 kVp, 40 μA; source-to-sample distance = 10 cm; sample-to-detector distance = 190 cm; exposure time 200 s.

The next example is for a small piece of carbon-fiber paper (Toray). This paper is used in the electrodes of fuel cells [130] and has excellent conductivity, very high strength, excellent gas permeability, a shape-memory property, and good (electrochemical) corrosion resistance. The PCX image, shown in Figure 4, was recorded with the X-ray source operated at 30 kVp, 40 μA, a source-to-sample distance of 10 cm and a sample-to-detector distance of 190 cm (experimental magnification of 20). The exposure time was 200 s. Individual carbon fibers (diameter of the order of 10 μm) are clearly visible, with phase contrast being the dominant mechanism.

**Figure 5:**
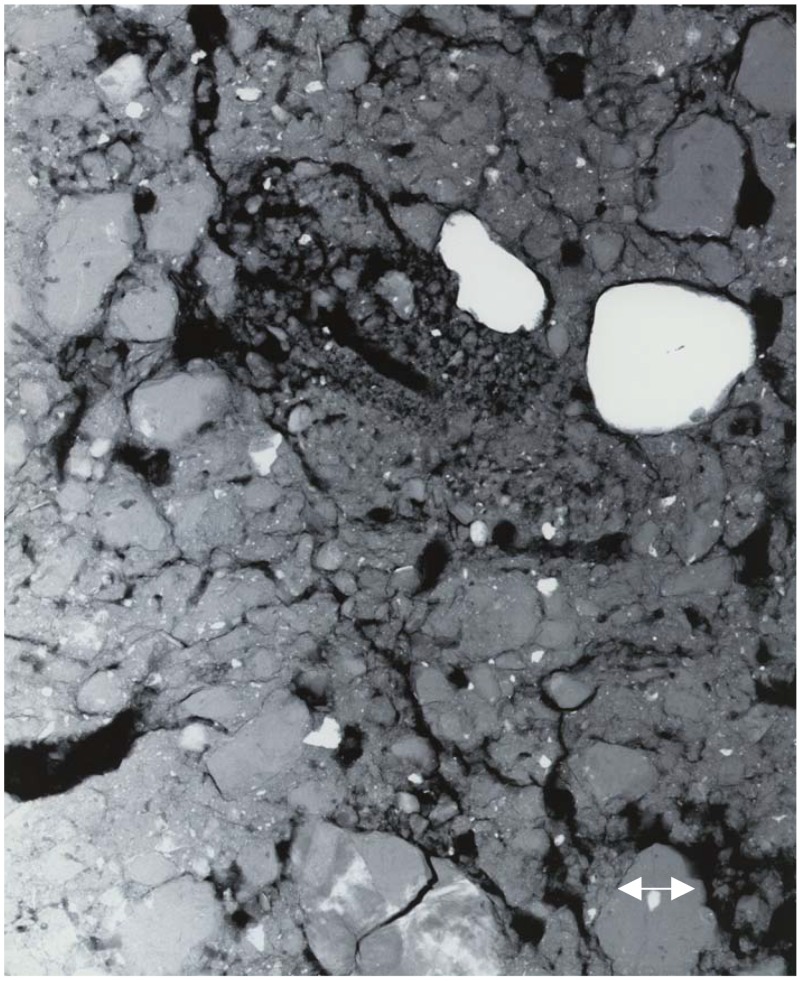
PCX image of a cross-sectional soil sample (a few mm thick): 30 kVp, 40 μA; source-to-sample distance = 10 cm; sample-to-detector distance = 90 cm; exposure time 60 s.

Finally, in Figure 5, we present a PCX image of a cross-sectional soil sample. Such images have been used to map the fine root structure of certain plants in order to study mechanisms for nutrient uptake [131]. This image was recorded with the X-ray source operated at 30 kVp, 40 μA, a source-to-sample distance of 10 cm and a sample-to-detector distance of 90 cm (experimental magnification of 10). The exposure time was 60 s. This particular image serves to demonstrate one of the extraordinary properties of image plates: their huge dynamic range. Whilst certain features in the image appear to be totally white or totally black, the raw image does actually contain considerable detail in these regions, but it is not possible to present the full wealth of information present, in a single representation of the data; we have thus chosen a representation which shows some of the finer details to best effect.

#### 3.1.3. PCX—Three-Dimensional Imaging (Micro-Tomography)

**Figure 6:**
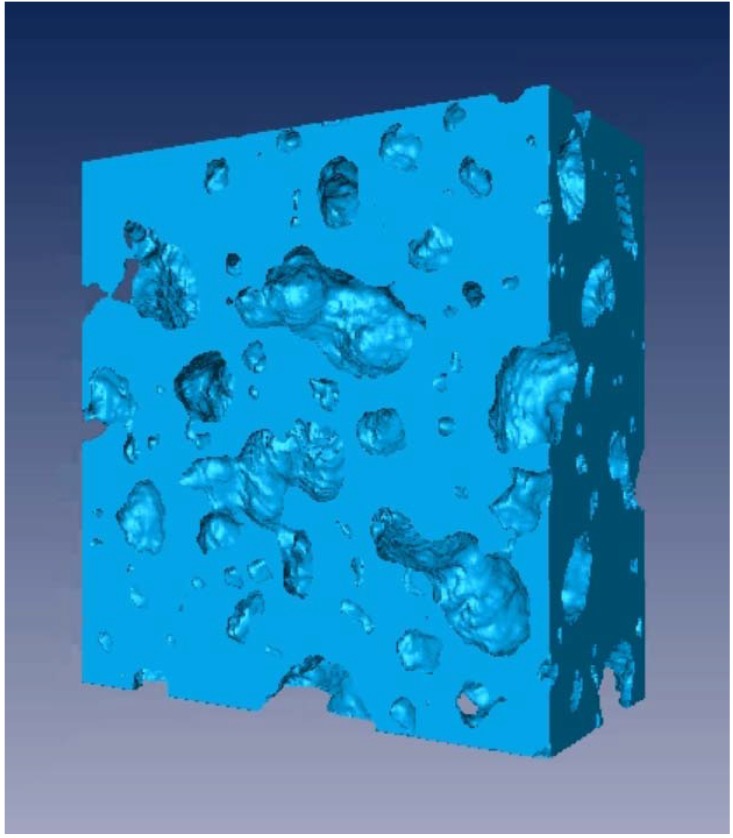
Rendered view of reconstructed PCX tomography data for a small (maximum side length ~1 cm) sample of HySSIL light-weight, high-strength concrete: 60 kVp, 25 μA; source-to-sample distance = 31 cm; sample-to-detector distance = 19 cm.

One of the products developed by HySSIL Pty Ltd., incorporating CSIRO research and development, is a light-weight, high-strength concrete [132]. This concrete has a high degree of porosity and an important aspect of characterizing this material is visualizing, non-destructively, this porous nature in three dimensions. For this application we used PCX tomography, with the microfocus X-ray source described above. However, in order to collect multiple images for a tomographic data set, it is not practical to employ image plates and we used a CCD. This Peltier-cooled, hard X-ray CCD was supplied by XCAM Ltd. and has 2048 × 2048 13.5 μm pixels. There is a fiber-optic screen hard-bonded directly onto the CCD plus a (field-replaceable) 3 mm thick fiber-optic face-plate coated with a 200 μm layer of structured CsI. The data was collected with the X-ray source operated at 60 kVp, 25 μA, a source-to-sample distance of 31 cm and a sample-to-detector distance of 19 cm (experimental magnification of 1.6). Multiple images (5 × 80 s exposure time) were collected at each angular step (2° intervals over 360°) together with flat-field and dark-current images. This raw data was pre-processed and then cone-beam tomographic reconstruction was performed, using the software package X-TRACT [133]. A rendered view of the reconstructed data is shown in Figure 6. The smallest pores are of the order of 100 μm in size. Such three-dimensional data provides the opportunity to not only quantify the degree of porosity as a whole, but also obtain information on the size distribution, connectivity and geometry of pores.

### 3.2. Higher-Resolution X-Ray Microscopy and Micro-CT Using the XuM

The XuM is a high-resolution X-ray microscope, based around a scanning electron microscope (SEM) [134]. This instrument generates X-rays by focusing the electron-beam of the SEM onto a metal foil target (usually tantalum), generating an X-ray source down to 100 nm in size, depending on the target material. X-rays from this source pass through a sample and form an image on detector water-cooled Princeton instruments LCX detector. This is based around a 1300 × 1340 pixel deep-depletion CCD working in direct detection mode. The maximum accelerating voltage of the microscope is around 30 kV, which, together with the sensitivity range of the CCD, sets the useable X-ray range at 3–15 keV. The instrument is most typically used between 10× and 100× magnification, and since the pixel size of the CCD is 20 μm, has much higher potential resolution for imaging than a typical microfocus source. The relatively soft X-ray energies coupled with the very small source size make this an ideal instrument for in-line phase-contrast imaging. A typical image acquisition time is around a minute, however, higher quality 2D images are created by summing 5–10 such images. Tomographuc data collection typically consists of 720 images of one minute exposure time, with a total data collection time of around 14 h.

The following section outlines three applications of in-line phase-contrast using the XuM to different materials characterization tasks, drawing out different aspects of phase contrast.

#### 3.2.1. Crack in Carbon Coating on Multi-Layer Coated Zirconia Sphere

As noted above phase-contrast imaging is very sensitive to cracks, voids and boundaries, as these features all represent a sharp change in X-ray refractive index in the sample, which gives rise to phase-contrast fringes in the image. 

**Figure 7:**
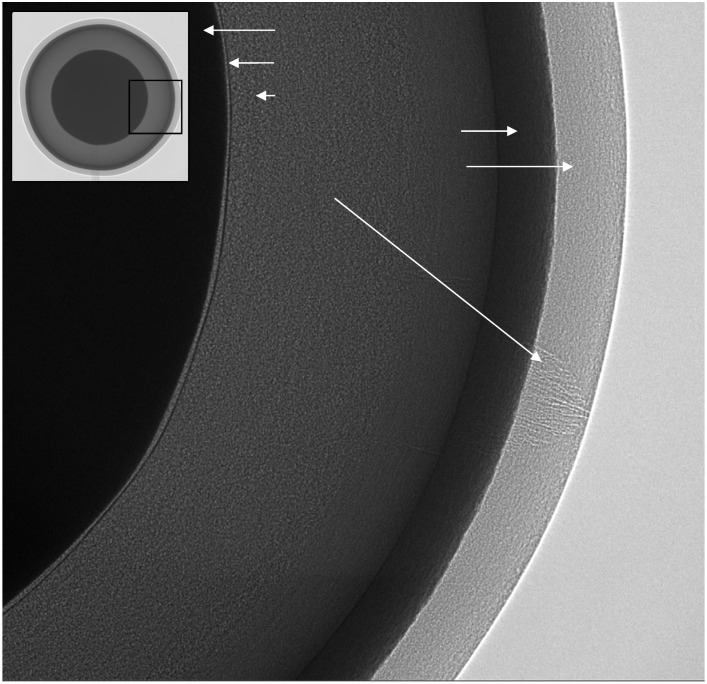
2D Phase-contrast X-ray image of a fuel pellet consisting of layers of pyrolitic carbon and silicon carbide on a zirconia core. In the main image a crack can be seen, highlighted by phase-contrast, in the outer pyrolitic carbon layer. The inset is a lower magnification image providing an overview of the whole sample, approximately 800 μm in diameter.

Figure 7 shows an XuM image of a multi-layer fuel-pellet model consisting of a zirconia core coated with a thin buffer layer followed by two pyrolitic carbon layers separated by a layer of silicon carbide. This type of sample is a model for TRISO fuel particles used in nuclear applications in which the particle core would be uranium dioxide [135]. The edge enhancement of the phase contrast makes the boundaries clearly visible, including very low-contrast boundaries like that between the buffer layer and the pyrolytic carbon, which shows some unevenness in the thickness of the buffer layer.

The image also highlights the presence of a fine crack in the outer pyrolitic layer, which was possible to determine (by rotating the sample) as being restricted to this layer only. Although the outside of the sample would be amenable to SEM analysis which might detect a crack, X-ray phase contrast gives much greater information on the penetration of the crack into the internal structure and the integrity of the internal layers. This is an example where a limited number of 2D images is sufficient to determine the quality of the layers in the sample. For more complex structures tomographic methods are typically required.

#### 3.2.2. Fibrous Tissue Scaffolds—3D Characterization of Almost X-ray Transparent Materials

In addition to sensitivity to cracks and voids, phase contrast is much more senstive to low-density materials than conventional methods. This is demonstrated below in the case of a study of the different morphology of fibrous tissue scaffolds for cell growth. This type of material is being actively researched for tissue engineering and repair [136,137]. Three such structures were examined: Two consisted of a loose mesh of different densities composed of fibers in the range of 20–25 μm diameter, while the third was a finer electro-spun mesh with fibres ranging from approximately 10 μm to as small as a micron across. These were all scanned in the XuM using a tantalum foil target for X-ray generation, giving an X-ray spectrum dominated by 8 keV X-rays. Tomographic data were acquired for each sample.

**Figure 8:**
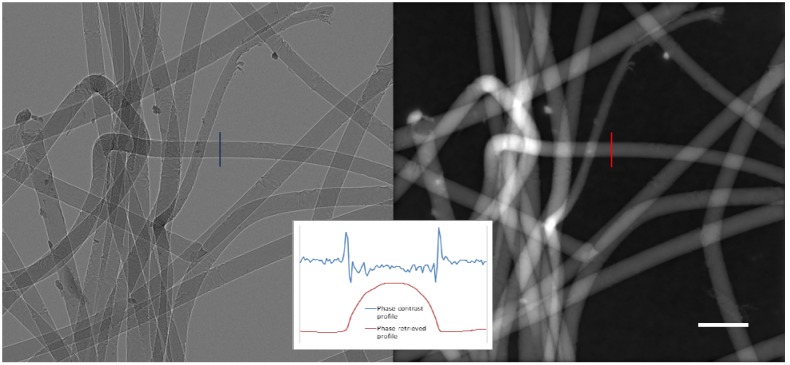
Left, close up phase-contrast image of tissue scaffold consisting of polymer fibers approximately 20 μm in diameter; right, the effect of phase-retrieval applied to the left hand image; inset, line profiles through the same fiber in each image.

Figure 8 shows a comparison between a raw 2D phase-contrast image of the first sample at high magnification, and the image after it has been processed by phase-retrieval using Paganin’s algorithm [59]. The inset to the figure shows line profiles through the same fibre in each image. The profile from the phase-contrast image shows how strongly the contrast is dominated by the fringes at the edge of the fibre, and how weak the absorption contrast is relative to the noise in the image. The profile from the phase-retrieved image corresponds much more closely to the expected thickness profile and has much better signal to noise. 

For phase-contrast tomography as in this case, the raw phase-contrast images are usually processed by phase-retrieval prior to tomographic reconstruction. This result is a far cleaner reconstruction with much improved signal to noise characteristics that is much more amenable to 3D rendering for data exploration and image segmentation for quantitative analysis. Reconstruction can be done without phase retrieval in which case the edge-enhanced nature of the images is preserved in the 3D reconstruction. This can be valuable to highlight cracks or voids, however it also can enhance certain types of tomographic artefacts, such as those arising from rotation stage misalignment, and it is also problematic for image segmentation. 

**Figure 9:**
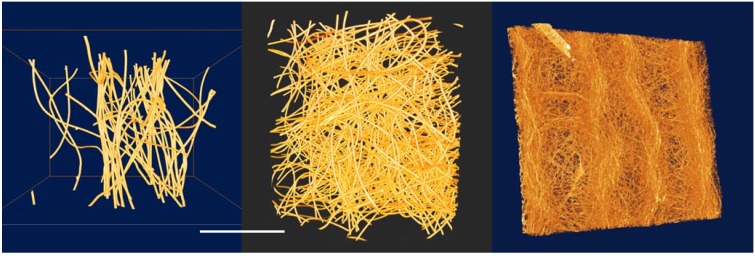
3D rendered views of tomographic datasets for each of the three scaffolds, the electro-spun scaffold is on the right and is ~100 μm thick. The width of the samples is approximately 2 mm in each case.

Figure 9 shows 3D rendered views of each of the three samples produced using Avizo^®^ software. The workflow incorporating phase-contrast imaging, phase-retrieval and tomographic reconstruction has produced high quality data suitable for quantitative analysis. As these samples are so weakly absorbing of X-rays, absorption contrast would have been insufficient to produce high-quality data. This is particularly the case for the electro-spun sample in which the finer fibers would have been completely invisible in an absorption-contrast image.

#### 3.2.3. Self Healing Polymers—The Complementary Nature of Phase and Absorption Contrast

Self-healing materials contain components, which allow them to self-repair cracks as they occur improving the lifetime of the materials. In this study we investigated a self-healing PMMA-PS composite polymer material, which incorporated capsules of dichlorobenzene (DCB) solvent approximately 60 μm in diameter. If the polymer cracked, the solvent was released from the ruptured capsules and dissolved the surrounding polymer in the crack region, enabling it to flow into the crack and repair it. The effectiveness of the healing process was investigated by producing cylinders of the materials, approximately 2 mm in diameter, cracking them and allowing them to heal.

Two samples were scanned using X-ray phase-contrast tomography in the XuM using a tantalum foil X-ray target generating X-rays with an average energy of 8 keV. One was scanned seven days after healing and another after three months. 

**Figure 10:**
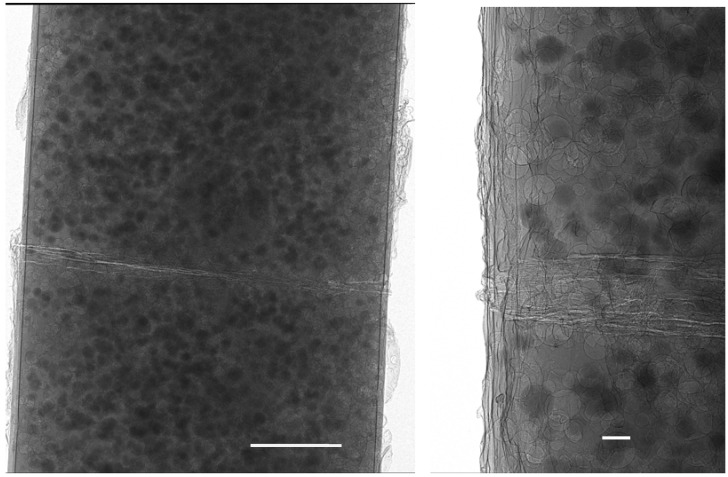
2D X-ray phase-contrast images of the partially healed crack in a cylinder of a polymer. The solvent capsules appear as dark circular regions due to X-ray absorption whilst the edges of the empty capsules and the crack are highlighted by phase-contrast.

Figure 10 shows 2D images of the three-month sample in the region of the crack. The solvent capsules are visible due to absorption contrast as the DCB solvent is of a higher density than the polymer; a number of empty capsules and the partially-healed crack itself are made visible through phase contrast which enhances the edges of these features.

**Figure 11:**
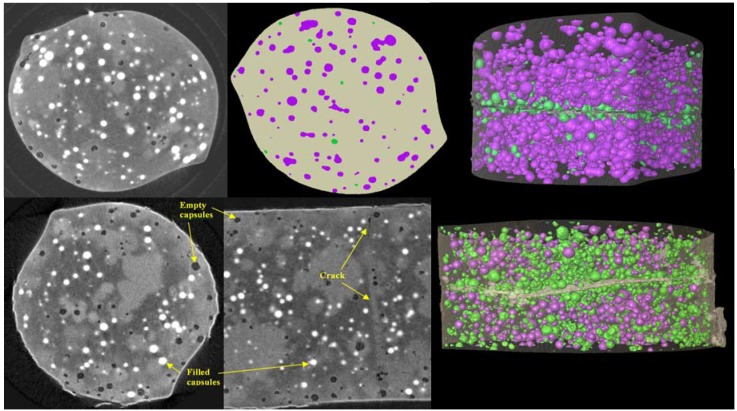
Upper row; a tomographic cross section, a segmented cross section and a 3D rendered view of the seven-day healed sample showing full capsules as purple and empty ones as green (sample diameter 2 mm). Note the band of empty capsules around the crack. Lower row; two tomographic sections in different orientations and a 3D rendered view of the three-month healed sample. Note the much higher proportion of empty capsules in regions away from the crack relative to the seven-day healed sample.

Phase retrieval was applied to the raw data prior to tomographic reconstruction. This resulted in data of sufficient quality for quantitative analysis. The reconstructed data was segmented using Avizo^®^ software which initially was used to separate the materials into empty capsules, capsules full of solvent, polymer matrix and the crack. This analysis showed that, as expected, capsules in the vicinity of the crack had ruptured and partially healed the crack region. As the plane of the crack was approximately horizontal this could be quantified by plotting the average volume of full *vs.* empty capsules running down the vertical axis of the sample. Figure 11 shows tomographic cross sections and 3D rendered views of the two samples. The rendered views clearly show the absence of full capsules in the plane of the crack, and also the larger number of empty capsules in the aged sample.

A secondary component of the analysis was focused on the effect of aging on the sample. This followed from the observation that although both samples had many more empty capsules in the vicinity of the crack, the three-month old samples showed significantly more empty capsules in non-crack regions than the seven-day sample. The empty capsules appear to coincide with denser regions of polymer that occur where solvent has been released, as can be observed in the healed crack. To quantify this qualitative observation and to compare the surface contact area of each type with full *versus* empty capsules, the data for the three-month old sample was further segmented to separate denser and less dense polymer. This confirmed that the denser polymer regions corresponded to empty capsules and that spontaneous rupture of capsules was an issue with aging in this material. Further details on this study can be found in papers by Mookhoek and co-workers [138,139].

## 4. Conclusions and Future Directions

In the 17 years since it was first demonstrated with hard X-rays, in-line phase-contrast imaging and tomography has demonstrated its worth in a wide variety of applications in materials science. A particular advantage of the technique compared to some other phase-contrast methods is that it is applicable in a laboratory setting using standard polychromatic X-ray sources. Furthermore it is very simple to implement and in some cases only one image is required to perform phase-retrieval. This means that in-line phase-contrast in a synchrotron setting can readily be applied in high-speed tomography applications, or applications requiring sample environments or complex stages for *in situ* experiments. 

Operating in edge-enhanced mode is now routine at many synchrotron micro-tomography beamlines. The laboratory-based micro-CT community has not yet taken up the use of phase-contrast methods to the same extent, however usage is growing. Micro-focus X-ray sources with source sizes down to 5 μm have been available for a number of years, but smaller source-sizes down to below a micron in X-ray sources are now available commercially for high-resolution micro-CT. Other exciting developments in the area of laboratory-based microfocus X-ray sources include rotating-anode and liquid-target versions, offering the promise of higher fluxes without an associated increase in source size. Alternatively the use of high-resolution detectors, such as in systems built by Xradia, enables higher-resolution imaging than the source size whilst still retaining good phase contrast. With such sources and detectors, phase-contrast imaging is much easier to achieve, and in some cases, where lower energies are being used, is almost unavoidable. In the light of this, in-line phase-contrast X-ray imaging and tomography may become as routine in the lab as it is increasingly becoming at synchrotrons.
